# Magnetic resonance imaging of the urethra

**DOI:** 10.1590/0100-3984.2023.0084

**Published:** 2023

**Authors:** Gustavo Fiedler, Leonardo Kayat Bittencourt, Christopher Zhou, André Guilherme Cavalcanti, Suzan Menasce Goldman

**Affiliations:** 1 Department of Diagnostic Imaging, Escola Paulista de Medicina da Universidade Federal de São Paulo (EPM-Unifesp), São Paulo, SP, Brazil.; 2 Department of Radiology, University Hospitals Cleveland Medical Center, Cleveland, OH, USA.; 3 School of Medicine, Case Western Reserve University, Cleveland, OH, USA.; 4 Universidade Federal do Estado do Rio de Janeiro (Unirio), Rio de Janeiro, RJ, Brazil.

## INTRODUCTION

The study of the urethra by dynamic magnetic resonance imaging (MRI) is an effective method for evaluating urethral morphology and functional appearance, as well as the adjacent pelvic organs and structures. The advantage of dynamic assessment is that it allows high-definition cine visualization of the multiple compartments of the pelvic floor, together with periurethral structures, thus enabling the diagnosis of pathologies and the determination of their effects on voiding.

## PROCEDURE

This study involved three male patients diagnosed with urethral stricture, including one case newly diagnosed via urethrocystography (case 1), one case of recurrent stricture (case 2), and one case of stricture developing after radical prostatectomy (case 3), as previously described^(1,2)^. In addition, we included patients who underwent MRI at the Diagnostic Imaging Center Clinic, part of the Diagnósticos da America SA group, in the city of Rio de Janeiro, Brazil, and who subsequently underwent urethroplasty. Patients with concurrent neoplasia or non-urethral stricture-related infravesical obstruction were excluded. The study was approved by the local institutional review board via Plataforma Brasil (Registration no. IRB 4.227.628), and all participating patients gave written informed consent.

### Technique

Dynamic magnetic resonance urethrography (MRU) was performed as previously described^(3)^. Before the examination, patients undergo peripheral venous access insertion and stimulation of diuresis with 500 mL of saline. In addition, 20–40 mL of lidocaine gel is applied to the urethral meatus. Patients lie on a stretcher during the examination.

For all patients, the examination was conducted in a 1.5-T scanner (Aera; Siemens Healthineers, Erlangen, Germany), with a standardized protocol^(4)^. The MRU protocol includes various sequences: axial T1-weighted urography; axial and sagittal T2-weighted sequences; coronal single-point gradient-echo sequences; sagittal maximum intensity projection images; and additional T2-weighted sequences acquired at rest and during straining. In some cases, sagittal T1-weighted images are acquired with and without gadolinium to enhance visualization. This noninvasive MRI technique assesses urethral anatomy, identifies strictures, and determines their extent, aiding diagnosis and surgical planning^(5,6)^.

### Aspects of MRU

The advantages and disadvantages of MRU are as follows^(7,8)^:

- It diagnoses urethral strictures and assists in surgical planning.
- It offers crucial anatomical insights for surgical decision-making.
- It assesses treatment effectiveness and procedurerelated recurrences.
- It provides detailed images of the urethra for more precise diagnoses.
- It is noninvasive, thus precluding the need for invasive procedures like urethrography or urethroscopy.
- It improves surgical planning by providing detailed anatomical data, thus improving accuracy.
- The cost of the procedure can limit access to it in some health care settings.
- In certain regions, the availability of the procedure is limited, which can delay diagnosis and treatment.
- Contrast (gadolinium) might be needed, which can pose challenges for patients who are allergic or have impaired kidney function.

## RESULTS

**Figure 1:**
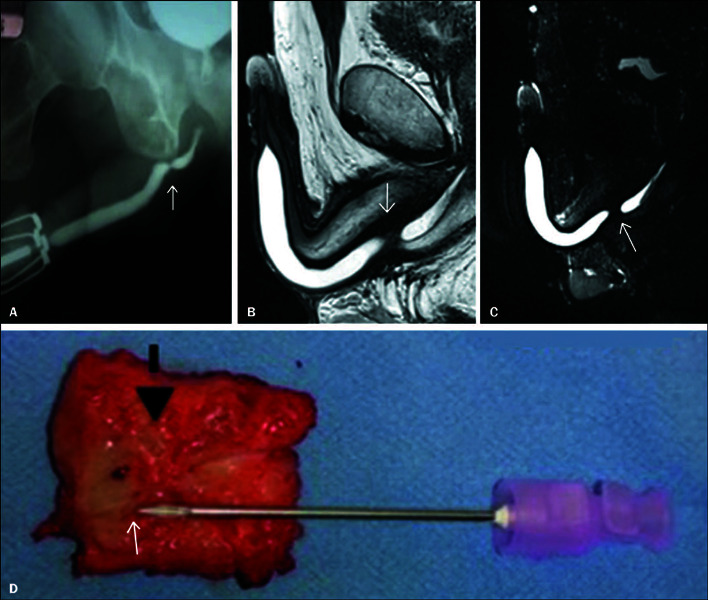
A: Retrograde urethrography demonstrating one area of narrowing, an annular stenosis in the distal posterior urethra (arrow). The annular stenosis permits retrograde filling of the bladder, as identified through multimodal analysis of stricture using retrograde urethrography and dynamic MRU. **B,C:** Sagittal T2-weighted images showing a bulbar segmental stricture with surrounding fibrosis, characterized by a hypointense signal surrounding the irregular stenotic section in the bulbar urethra (arrows). **D:** Resected stenotic segment of urethra showing good correspondence with the images acquired by retrograde urethrography and dynamic MRU.

All three patients showed bladder neck opening during voiding (Table 1). Intraoperative and MRU findings consistently aligned regarding stenosis characteristics. Dynamic MRU also accurately identified single and multiple stenoses. Notably, in case 1, spongiofibrosis (partial penile segmental stenosis), characterized by a hypointense signal and contrast uptake on T2-weighted images (Figure 1, A–C), was observed after removal of the stricture. The fibrostenosing aspect, with the margin as a potential area for disease recurrence, can be seen in Figure 1D.

**Table 1:** Clinical and demographic characteristics of patients with urethral stricture.

Variable	Case 1	Case 2	Case 3
Etiology	Sexually transmitted infection	Sexually transmitted infection	Iatrogenic (postoperative)
Stenosis	Fixed	Fixed	Fixed
Opening of the bladder neck	Present	Present	Present
Stenosis thickness	1 mm	3 mm	6 mm
Stenosis length	26 mm	3 mm	20 mm
Location	Penis	Bulbar urethra	Bulbar urethra
Distance from the stenosis to the glans	12.9 cm	16.0 cm	17.0 c m
Current smoker	Yes	Yes	Yes

## CONCLUSION

Knowing whether or not the bladder neck opens during voiding facilitates the surgical planning. The fact that intraoperative and MRI findings fully aligned on stenosis characteristics attests to the accuracy of dynamic MRU, which reliably detected single and multiple stenoses, as well as post-procedure spongiofibrosis.
